# Heavy Metals in Foods and Beverages: Global Situation, Health Risks and Reduction Methods

**DOI:** 10.3390/foods12183340

**Published:** 2023-09-06

**Authors:** Elena Cristina Scutarașu, Lucia Carmen Trincă

**Affiliations:** Faculty of Horticulture, “Ion Ionescu de la Brad” Iași University of Life Sciences, 3rd M. Sadoveanu Alley, 700490 Iași, Romania; cristina_scutarasu@yahoo.com

**Keywords:** heavy metals, health concerns, contamination, industrialization, food safety

## Abstract

Heavy metals are chemical elements with a toxic effect on the human body. The expansion of industries has led to significant increasing levels of these constituents in the environment. Intensive agriculture can also lead to an increased concentration of heavy metals as a result of using different fertilizers and pesticides. Heavy metal accumulation in soil and plants represents a serious issue because of the potential risks to consumers. There are several methods available for the removal of these toxic components from different substrates (chemical precipitation, electrodialysis, coagulation and flocculation, photocatalytic removal, and adsorption-based processes), but most procedures are expensive and difficult to perform. Thus, more research is needed on the development of low-cost methods in foods. This work represents a review on the heavy metal presence in different food substrates (such as fruits and vegetables, milk and dairy products, meat and meat derivatives, oils, and alcoholic beverages) and provides an overview of the current situation worldwide, taking into account the fact that risks for human health are induced by the intensification of industry and the high degree of pollution. Considering that the toxicological quality of food affects its acceptability, this work provides valuable data regarding the actual situation on the proposed topic.

## 1. Introduction

Heavy metals are elements with an atomic weigh over 63.5 and a specific gravity higher than 5.0 that are generally dangerous to human health and the environment. The major elements included in this class are as follows: lead—Pb; cadmium—Cd; cobalt—Co; chromium—Cr; copper—Cu; iron—Fe; arsenic—As; nickel—Ni; zinc—Zn; and mercury—Hg [1]. The development of industry, the excessive use of chemical substances in agriculture, as well as the intensification of car traffic, in addition to the multiple benefits brought to humanity, continuously produce great ecological imbalances, sometimes reversible, sometimes irreversible, with a particular impact on the quality and safety of food, food resources, and the health of consumers. Heavy metals can originate from both natural (rocks) and anthropological sources (metal mining, smelting, trash dumping, incineration, pesticides, etc.) [2]. So, with the industrial revolution and economic globalization, the numerous environmental contaminants have significantly increased. These contaminants do not dissolve, and they accumulate in the environment [2]. The diverse and emerging food security issues have become a global concern [3,4]. Traces of different heavy metals were found in food (vegetables, meat, fish, milk, etc.) due to their mobility and bioaccumulation in water sources. By whatever means heavy metals enter food and beverages, once they enter the body, they are oxidized and form stable bonds to enzymes or protein molecules. This results in dysfunction, abnormalities, or even damage [5]. Nowadays, there is a growing preoccupation regarding the heavy metal contents in food and beverages and their subsequent consequences for the daily diet and human life chain safety.

**Figure 1:**
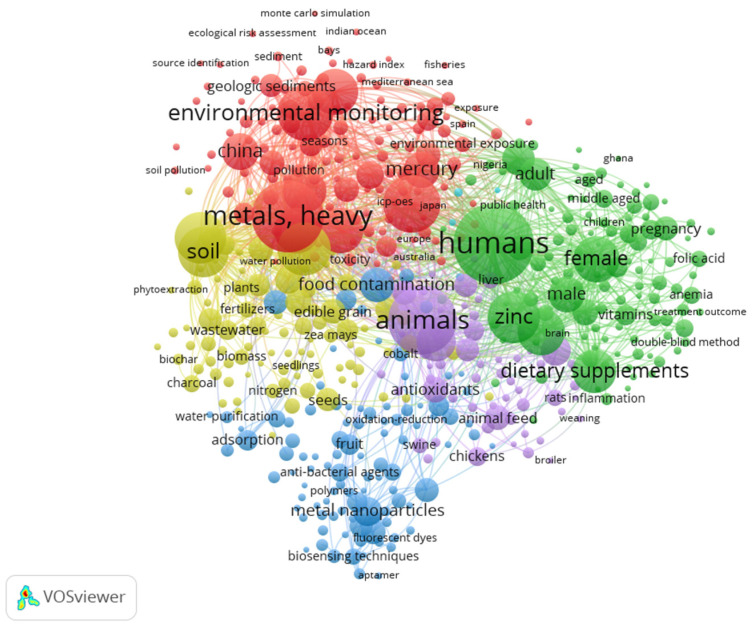
Co-occurrence of the most common 500 keywords of the existing research on heavy metals topics. The size of node represents the number of occurrences of a keyword, and the length of the connection between two nodes represents their relationship.

**Figure 2:**
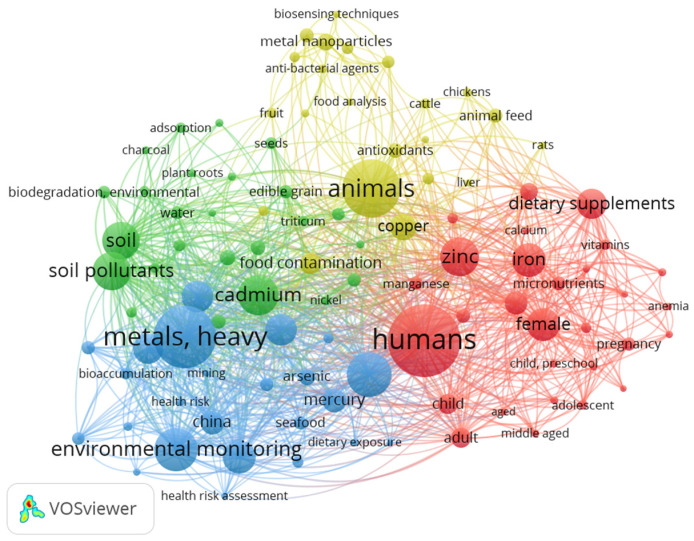
Co-occurrence of the most common 100 keywords of the existing research on heavy metals topics. The size of node represents the number of occurrences of a keyword, and the length of the connection between two nodes represents their relationship.

Several studies have focused on the concentrations of heavy metals in food [6,7,8]. In Figure 1 and Figure 2, maps are shown that were created using bibliographic data from PubMed database files. For the subject “heavy metals in food”, a total of 35,945 papers were published in the last 5 years. In Figure 1, the 500 most common keywords of the existing research are represented. For a better visualization, in Figure 2, the most common 100 keywords were carefully chosen.

From Figure 1 and Figure 2, it can be highlighted that the most-studied subjects refer to the prevalence of heavy metals in the environment and how they affect the animal and human body. Most human research has focused on women and how heavy metal contamination affects their fertility and pregnancy. Globally, most studies were found in China. Heavy metal contamination is mainly determined by the high level of pollution existing in this area.

Among the studies found, the contamination of water and food with cadmium, copper, mercury, zinc, and iron are among the most frequently analyzed subjects. The existing literature focuses on the presence of heavy metals in cereals, seeds, fruits, and seafood. Metals such as zinc and iron are indeed used as dietary supplements, especially for women during pregnancy or with children.

To reduce food contamination, international organizations (the Food and Agriculture Organization of the United Nations—FAO; the World Health Organization—WHO; and the International Organization of Wine and Wine—OIV) have established limits for these chemical components in food. For example, in Codex Alimentarius [9], the Official Journal of the European Union (Reg. EC 1881/2006), the International Code of Oenological Practices [10], and the Compendium of International Methods of Wine and Must Analysis [11], limits for some heavy metals are put forward. Of these, arsenic, lead, mercury, and cadmium have been prioritized in governmental programs for monitorization and reduction. Arsenic should be found at a maximum of 0.1 mg/kg in edible fats and oils, fat spreads, and blended spreads; at a maximum of 0.01 mg/kg in natural mineral waters; at a maximum of 0.35 mg/kg in husked rice and 0.2 mg/kg in polished rice; and at a maximum of 0.5 mg/kg in salt and food-grade. Cadmium is limited to 0.01 mg/L in wine; 0.05 mg/kg in brassica vegetables, bulb vegetables, and fruiting vegetables; 0.2 mg/kg in leafy vegetables and wheat; 0.1 mg/kg in legume vegetables, pulses, root and tuber vegetables, and stalk and stem vegetables; 0.4 mg/kg in polished rice; 0.003 mg/kg in natural mineral waters; 0.5 mg/kg in salt and food-grade; 0.8 mg/kg in chocolate containing more than 50% cocoa and 0.9 mg/kg in chocolate containing more than 70% cocoa; and 2 mg/kg in marine bivalve mollusks and cephalopods. Copper is limited to 1 mg/L in wine and 2 mg/L in liqueur wines obtained from unfermented or slightly fermented grape juice. Regarding lead concentrations in food, the following maximum limits are established: 0.01 mg/kg in infant products and natural mineral waters; 0.02 mg/kg in milk and dairy products; 0.04 mg/kg in grape juice; 0.1 mg/L in wine (starting from the 2019 harvest year); 0.15 mg/L in liqueur wine (from the 2019 harvest year) and in fat spreads and blended spreads; 0.05 mg/kg in fruiting vegetables, preserved tomatoes, canned chestnuts, canned chestnut puree, and berry fruit juices; 0.1 mg/kg in some fruits (berries, pome fruits, stone fruits, pineapple, avocado, mango, and canned fruits), brassica vegetables, bulb vegetables, legume vegetables, pulses, root and tuber vegetables, canned vegetables, pickled cucumbers, cattle meat, pig meat, poultry or sheep meat, and wine; 0.2 mg/kg in cranberry, currants, elderberry, cereal grains, and fortified wines; 0.3 mg/kg in fish; 0.3 mg/kg in fresh farmed mushrooms, shiitake mushrooms, and oyster mushrooms; 0.4 mg/kg in jams, jellies, marmalades, mango chutney, and table olives; 0.1 mg/kg in oils and edible fats; and 1 mg/kg in salt and food-grade. Mercury is limited to 0.001 mg/kg in natural mineral waters and 0.1 mg/kg in salt and food-grade. This compound can also be found in a combined form, as methyl mercury, which is limited to 1.2 mg/kg in tuna, 1.5 mg/kg in alfonsino, 1.7 mg/kg in marlin, and 1.6 mg/kg in shark meat. Zinc is limited to 5 mg/L in wines.

## 2. Sources and Health Concerning

Heavy metals are potentially toxic in high amounts or after long-term exposure [12]. These elements manifest the ability to move and concentrate in different organs, leading to various health issues [13]. For example, lead affects the normal activity of enzymes and is linked with carcinogenesis, mutagenesis, and teratogenesis in experimental animals [14]. Lead poisoning is still a major public health risk, especially in developing countries [15]. The toxic action of lead is influenced by the chemical form in which the element is found, being absorbed in the body in a proportion of about 90%. Initially, the manifestations are non-specific, with Burton’s line present (a blue-gray area on gums) [16]. Organic lead accumulates mainly in the bones, producing various transformations of the hematoforming marrow, and less in the nervous system, organs (liver, kidneys), or muscle tissue. Inorganic lead is absorbed from the intestinal tract, more so in children (around 40–50%). Of the total absorbed lead, most of it is eliminated through urine (about 75%) and about 16% through feces. The most dangerous entry route is considered the respiratory one, as lead reaches the blood directly, without the possibility of elimination through urine or feces. Lead poisoning begins with anemia, caused by a reduction in the life span of erythrocytes and a decrease in hemoglobin synthesis. Disorders of the central nervous system appear later, such as hyper reactivity, impulsive behavior, changes in perception, and decreased learning capacity. In the peripheral nervous system, claw-hand disease can develop. In acute forms, irritability, restlessness, muscle tremors, headaches, ataxia, unclear consciousness, and memory loss may occur. In the last stage, renal failure, convulsions, coma, and finally death are observed. However, lead poisoning following the consumption of food is rare; the cases reported so far have had the source of alcoholic beverages (liqueurs, cider wine) kept for a long time in ceramic vessels varnished with varnishes containing lead or fraudulently distilled beverages by using car radiators [14,17].

The widespread use of cadmium in industries has caused an important ecotoxicological problem. It is freely found in living organisms such as clams, crustaceans, mushrooms, etc. Following the consumption of foods with a high level of cadmium, associated with an irrational diet and insufficient intake of vitamin D, osteomalacia (soft bones) can occur [18]. Cadmium is a cumulative toxicant; its action is exerted on both the urinary and respiratory systems [19]. Cadmium is easily absorbed by plants, with the highest concentration found in fungi. In the case of animals, it is absorbed in the liver and kidneys. The main foods contaminated with this element are pork, fish, potatoes, milk, and beer. It does not accumulate in eggs, while seafood can present significant amounts of cadmium. The concentration of cadmium absorbed by the human body following food ingestion is between 5% and 16%, the value doubling under the influence of Ca^2+^, protein, or Zn^2+^ deficiencies. Cadmium is absorbed in the liver, spleen, adrenal glands, and duodenum (in the first 48 h after ingestion). Accumulation in the kidneys occurs much more slowly, with the maximum level being reached after approximately 6 days. Cadmium is involved in high blood pressure [14]. This element is characterized by high stability in the body, the excretion is very slow, and the life span is estimated at 20–40 years. Lung and kidney functions are affected by increasing the excretion of glucose, amino acids, uric acid, and proteins; the liver is also affected by increasing gluconeogenesis, thus resulting in hyperglycemia. There is an increase in the circulating levels of epinephrine, norepinephrine, and dopamine in the brain. It has also been established that it has hypertensive and teratogenic effects and is considered to be one of the most powerful metallic carcinogens known to date. A diet rich in elements such as zinc, iron, selenium, copper, and ascorbic acid favors reducing or even eliminating the toxic effects of cadmium [17,20].

Copper presents a high degree of toxicity [21], being found in combined forms (coveline, chalcosine, chalcopyrite, etc.). This element is used in the metallurgical and paint industries, glass, ceramics, and the manufacture of phytosanitary products. An important source of contamination is the equipment used in processing, handling, and storing food. Intoxications can also be caused by medication, by using copper sulfate as an emetic or tenifuge, but such situations are very rare. Copper is considered an essential element for the body, and deficiencies in this element cause different disorders, such as Menkes disease (manifested by stunting and the appearance of severe neurological difficulties) or Wilson’s disease (copper is stored in the liver and in the central nervous system) [21]. The toxicity of copper is higher through skin penetration, the respiratory tract, or directly into the blood because it can be eliminated through vomiting from the digestive tract. Food poisoning is rare in the case of copper, occurring in situations where the food product is highly acidic and, following prolonged contact with the metal, a significant amount of metal is dissolved. Thus, foods kept for a long period of time in copper packaging will turn green and present a metallic taste. Copper sulfate and copper acetate show low toxicity [18]. In large doses, the hemolytic and irritating effects, as well as the keratolytic effect, are noted [22].

Zinc is an essential element that the human body can tolerate up to 2 mg/kg when administered for a long-term period [22]. This metal can be found in numerous food substrates, such as fish, red meat, grains, dairy products, cereals, etc. The elimination of zinc from the body is ensured by the excretion of feces and urine. The consumption of small amounts of zinc supplements by children would favor a significant increase in height and weight [23]. In high amounts, it causes digestive and metabolic disorders. Soluble zinc salts have, in contact with the body, a caustic action. High doses of zinc lead to digestive or metabolic disorders [22]. This element can cause paralysis of the central nervous system and damage to the circulatory system and muscles. Additionally, among the symptoms of zinc poisoning are anemia, growth stagnation, and comatose states [18].

Arsenic can be found as arsenite or arsenate, both of which are lethal to humans and animals. This element originates from natural sources (volcanic ash and geothermal springs) [24], agro-industrial treatments (herbicides, pesticides, and fungicides) [5], and electronics [24]. The toxicity of arsenic is usually determined by its ability to interact with the sulfhydryl groups of proteins and enzymes and to substitute the element phosphorus in biochemical reactions [25]. In a study conducted by Rahman et al. [26], children were the most affected, followed by females and males. In general, neurological complications are pronounced in the case of children [26]. Long-term exposure can affect the cardiovascular and reproductive systems and produce cancer or diabetes [27]. According to Rasheed et al. [24], most studies refer to the total arsenic content of food and beverages, and more studies are required for individual forms. The ingestion of arsenic from water is the main route of exposure [24].

Mercury is usually used in different industries, such as paint batteries, chloro-alkali production, in thermometers, fluorescent lights, and dental amalgams [5]. Mercury exists in many chemical forms (inorganic and organic mercury). The degree of toxicity varies according to the form and concentration in which mercury is found in the analyzed substrate. Thus, methylmercury is quickly absorbed through the intestine and is deposited in numerous tissues. This form does not cross the blood–brain barrier as efficiently as elemental mercury. In the brain, methylmercury is transformed into elemental mercury by demethylation. Mercury and mercury salts affect internal organs (especially the intestinal mucosa and kidneys), while methylmercury is distributed throughout the body [28]. The contamination by mercury was reported in fish, sediments, hair, blood, and urine [29]. This metal induces tremors and sleep disturbances, irritability, excitability, excessive shyness, muscular spasms, loss of memory, depression, etc. [24]. In a study conducted by Rasheed et al. [24], exposure of newborn children to mercury induced the loss of cognitive ability, severe mental problems, and psychomotor alterations.

Chromium can be found in different fertilizers, industrial equipment, textiles, and paper industries [5]. Its toxicity refers to allergic reactions, anemia, pathophysiological defects, burns, and respiratory and gastrointestinal cancers [30]. Chromium can be found in different forms (with chemical valences from 0 to 6), but the Cr(III) and Cr(VI) forms are usually stable. Large amounts of Cr(III) negatively influence the absorption of trace elements in animals and humans and affect neuronal functions and kidney and liver activity. In addition to the toxicity demonstrated in the human body, Cr(VI) is also more toxic to the environment, affecting soil fertility and plant development (it changes the pH, affects the enzymatic activities and photosynthesis of plants, causes oxidative DNA damage, and destroys some microorganisms) [31]. DesMarais and Costa [32] postulated that most of the existing studies have shown that the inhalation of chromate induces lung cancer. One of the principal pathways of human exposure to chromate is drinking water.

Cobalt is used in coloring glasses, pigment production, or the alloys industry [5]. This element accumulates in the human body and causes allergic contact dermatitis, asthma, hepatotoxicity, memory loss, optic atrophy, cardiovascular diseases, a reduction in fertility, etc. [33]. The first cases of cobalt toxicity were identified between 1950 and 1970 in the USA, associated with the consumption of beer in which cobalt sulfate was added as a foam stabilizer. They were represented by men, generally malnourished, who used to consume such beer in large quantities (over 20 L). The elimination of this metal is carried out mainly through urine. People who work in the heavy metal industry and carry out diamond polishing activities are significantly exposed to developing toxicity, especially lung disease. In their case, symptoms started with dyspnea, cough, and wheezing [34].

Nickel cans originate from electroplating activities, storage batteries, casting industries, nickel-plated jewelry, smoking cigarettes, and electrical pieces [5]. Acute toxicity symptoms may manifest as headaches, vertigo, tachycardia, sweating, and visual disturbances. In cases of chronic inhalation exposure, severe respiratory disorders were reported. Nickel exposure determines the formation of free radicals in the human body, the modification of DNA bases, and has carcinogenic effects [35]. According to Genchi et al. [36], nickel toxicity is influenced by mitochondrial dysfunction and oxidative stress. Epigenetic changes driven by nickel exposure disrupt the genome. In high proportions, nickel affects the metabolic activities of plants, the photosynthetic transport of electrons, and the biosynthesis of chlorophyll. Kitchen kettles with nickel-plated elements can release nickel into the water when brought to boiling temperature. Additionally, nickel nanoparticles find applications in various fields, including catalyst manufacturing, the production of magnetic materials, biological drugs, and additives for lubricants [36].

## 3. Methods of Analysis

Identification and quantification of heavy metals in food can be made using different methods, such as Inductively Coupled Plasma Atomic Absorption Spectrometry—ICP-AES [37,38,39]; Flame Atomic Absorption Spectrometry—FAAS [39]; Mass Spectrometry with Inductive Coupling—ICP-MS [40,41]; Graphite Furnace Atomic Absorption Spectrometry—GF-AAS [42]; High-resolution Continuum Source Graphite Furnace Atomic Absorption Spectrometry—HR-CS-GFAAS [43,44]; Anion Exchange Chromatography Coupled to Inductively Coupled Plasma Mass Spectrometry—AEC-ICP MS [45]; Microwave Induced Plasma Optical Emission Spectrometry—MIP OES [46]; Electrochemistry [47]; Liquid Chromatography coupled with Mass Spectrometry (LC-MS/MS) [48]; Gas-Chromatography—tandem Mass Spectrometry (GC-MS) [49].

Among those mentioned, FAAS and GF-AAS are often used in most analytical laboratories. FAAS allows for identifying and quantifying a lot of metals (Co, Cd, Cd, Cu, Fe, Fe, Mn, Ni, Pb, and Zn) that are found even at trace levels. This method is characterized by low operation costs and good analytical performance, but the main disadvantage is the limitation of mono-elemental detection and the range of linear responses [50].

Some authors have proven that the variant with ICP-AES constitutes the most powerful analytical tool (rapid analysis speed, high analytical sensitivity, high accuracy and precision, and wide measuring range) for evaluating the concentration of heavy metals in any food substrate or drink [38,39,50,51]. This technique involves using plasma from argon for atomization and involves high costs with the equipment and operating procedure. Additionally, precision and accuracy are diminished when the solid sample is directly analyzed and needs to be converted to a liquid phase [50].

The ICP-MS technique enables a multi-element analysis with a large analytical range of linear responses, a low detection limit, easy and simple sample preparation, high-resolution tandem mass-spectrometry, etc. However, the equipment and laboratory setup costs are high, involve a high level of expertise of the operators, and multiple high purity gases are needed [40]. Compared to ICP-MS, FAAS and GF-AAS techniques enable a single-element analysis, require a higher sample volume, have a limited analytical range, and involve the utilization of flammable gases [40].

Techniques based on atomic spectroscopy are considered efficient due to their high sensitivity, selectivity, and rapidity [39]. The value of the heavy metal concentration estimated by the various methods mentioned is dependent on the properties of the metal and its concentration in the analyzed substrate [51].

Achieving accurate results is conditional on choosing an appropriate digestion procedure and adapting it to the situation [51]. Two main steps must be taken into consideration for heavy metal determination in food and beverages: sample preparation (digestion) and quantification (actual determination) [51]. Considering that many of the existing analytical methods involve a sample preparation step (decomposition), many authors have focused on the optimization of the digestion step (with dry, wet, and microwave ash) [38,39]. In that sense, Scaeteanu et al. [51] recommended the utilization of the FAAS method for Zn and Fe; digestion with an aqua regia-HClO_4_ mixture is more effective for Cu [51], while the dry ashing method was confirmed to be suitable for lead evaluation. According to Scaeteanu et al. [51], X-ray fluorescence enables simultaneous analysis of many heavy metals without digestion or degradation of the substrate [50]. For methods using chromatography, the main limitations are the high cost of equipment acquisition and maintenance, high operational costs, limited sample throughput, large size and impossibility of portability, and the need to provide an infrastructure to maintain power, gases, and exhaust. Compared to conventional methods that require relatively long working times and the use of large amounts of reagents, biosensors have gained popularity in recent years due to their small size, low cost, ease of sample preparation and operation, and fast response [51].

## 4. The Presence of Heavy Metals in Food

Nowadays, the levels of heavy metals in food and beverages do not present severe risks to human health for many geographical regions. However, there is a growing trend of environmental contamination, underscoring the need for continuous monitoring of heavy metals to safeguard human health [52].

This paper focuses on existing studies that investigate the presence of heavy metals in common dietary staples, such as fruits and vegetables, milk and dairy products, meat and derivatives, edible oils, as well as certain alcoholic beverages like beer and wine, considering that these types of alimentary products are the main constituents of the daily diet in many regions. The concentrations of heavy metals in food are heterogeneous due to the numerous factors that can affect their accumulation.

### 4.1. The Presence of Heavy Metals in Fruits and Vegetables

Fruits and vegetables present high nutritional values, and their consumption in fresh form helps in the maintenance of health and the prevention and treatment of various diseases. Fruits and vegetables contain both essential and toxic heavy metals in different amounts [53]. Numerous studies were conducted to analyze the content of heavy metals in fruits and vegetables from various countries and regions, especially those with large industrial activity or a high level of pollution [54,55,56,57]. Some of their results are presented in Table 1. The authors investigate factors like soil contamination, agricultural practices, and environmental conditions that may contribute to heavy metal accumulation in fruits and vegetables. Spinach and lettuce are among the most studied vegetables regarding their heavy metal content [55,56,57]. Fruits like apples, bananas, grapes, and berries, which are widely consumed [53,58,59,60], are also often tested for their heavy metal levels. Higher values than those allowed by Codex Alimentarius [9] were reported in some cases. For example, As^3+^ and Zn^2+^ presented higher values in the lettuce samples from the USA and, respectively, the Romanian aliquots [61,62]. Moreover, Zn^2+^ is usually found in high levels in Romanian samples; Ni^2+^ is the main heavy metal in Egyptian samples (due to highly polluted soils), while Cu^2+^ is predominant in Chinese tested aliquots.

### 4.2. Milk and Dairy Products

Milk is a basic component of the daily diet and contains essential nutrients for the normal functioning of the human body. Several studies regarding the concentrations of heavy metals in milk and dairy products were conducted over time [61,61,68,69,70]. Some results are presented in Table 2. High levels of these compounds may be due to the exposure of animals to environmental pollution or the consumption of contaminated water and feed [71]. Pilarczyk et al. [72] demonstrated that heavy metal content in milk is influenced by a cow’s breed. The authors obtained significantly higher concentrations of Pb^2+^, Cd^2+^, Cu^2+^, and lower levels of essential minerals in milk from Holstein Friesian cows compared to samples from Simmental cows. Özbay et al. [61] highlighted the significant impact of feed and water on As^3+^ levels in milk. Besides that, the authors showed that seasonal changes can affect heavy metal levels in milk. In the study of Meshref et al. [62], lead levels exceeded the maximum allowed value (0.02 mg/kg) established by the Codex Alimentarius [9]. The presented studies emphasize different amounts of heavy elements, probably in accordance with the existent differences in the air quality of each geographical region and land uses for feeding animals [73]. In general, the analyzed milk and dairy products were poor sources of copper and zinc. It can be said that milk contributes only to a small extent to covering the body’s needs.

### 4.3. Meat and Meat Derivatives

Heavy metals in meat and meat products come from contaminated animal feeds, especially in regions with intense industrialization [76]. Some authors reported various amounts of heavy metals in meat and meat derivatives produced in different countries [52,76,77,78,79,80]. Hoha et al. [77] obtained higher values of heavy metals in pork salami and sausages obtained in Romania, compared to less processed samples like ham and bacon. The authors postulated that higher quantities of contaminated spices could be the main explanation. The concentrations of the analyzed metals were below the Codex Alimentarius limits [9], but their cumulative effect should be taken into consideration. Salim et al. [80] obtained values that exceeded the Codex Alimentarius [9] permissible levels in the case of Pb^2+^ and Cd^2+^ ions in red meat samples from Asia and Africa. The authors postulated that Cd^2+^ was found in higher concentrations in meat with a high fat content and was accumulated in fat tissues [80]. In a previous study, Wang et al. [14] highlighted some different amounts of heavy metals in meat products from China during 2015–2017, with chromium being found in higher amounts while mercury being the lowest.

The results (Table 3) highlight that meat consumption carries a health risk due to the high accumulation of heavy metals.

### 4.4. Edible Oils

Vegetable oils contain trace metals such as Fe^2+^, Cu^2+^, Ca^2+^, Mg^2+^, Co^2+^, Cd^2+^, and Mn^2+^, which can accelerate the rate of oil oxidation [81]. Heavy metals in oils may originate from the soil, primarily due to the intensive use of fertilizers and pesticides or from contamination during the manufacturing process [82,83]. Their occurrence in vegetable edible oils is a result of environmental contamination, refining processes, transfer during transport, or the packaging process. Even if the used seeds contain high amounts of metals, most of them are not extracted from the resulting oils [41]. Several papers have been published on this topic, presenting varying concentrations of heavy metals (Table 4) [41,84,85,86,87,88,89,90,91]. Of particular concern is the excessive presence of Pb^2+^ and As^3+^ (exceeding 0.1 mg/kg) found in many assortments of edible oils (olive, rapeseed, sesame, sunflower, corn, cottonseed, and soybean oils) from various countries (London, Pakistan, China, Iran, and Monte Carlo). The concentrations of heavy metals are influenced by factors such as plant species, variety, bioavailability, cultivation techniques, and environmental conditions, including the capacity of bioaccumulation [92]. Sunflowers and olive oils, in particular, tend to accumulate higher levels of Fe^2+^, Cu^2+^, and Zn^2+^ [93].

### 4.5. Alcoholic Beverages (Wine and Beer)

Most of the heavy metals present in alcoholic beverages can originate from agricultural pesticides or contamination from processing equipment (pipelines, containers, tanks, filtration systems, aluminum cans, etc.) [58,99].

Table 5 summarizes some of the existing research findings on this topic. In the beer industry, trace components, including heavy metals, represent approximately 0.02% of malt extract. This includes hazardous metals, which can be transferred from source ingredients to the final beer product and brewing residues. The concentrations of these metals depend on the chemical composition of the raw materials and their capacity to dissolve into the solution over the brewing process. Considering that beer is one of the most widely consumed beverages globally, regular consumption can have adverse effects on people’s health. Some researchers have noted that beer can contain varying levels of major, minor, or trace metals throughout the manufacturing process. Their presence can also affect the final quality of the beer in terms of flavor stability and haze [99]. Zuffal and Tyrell [100] highlighted that metal ions and peroxides led to the rapid development of stale aroma in beer, with heavy metals being essential in the beer aging process. The importance of manganese for beer flavor stability has also been confirmed by the authors. According to Hudson [101], these components are involved in beer haze formation.

In winemaking, heavy metal sources are similar to those in beer production. According to Han [66], heavy metals such as Cu^2+^ and Pb^2+^ can be absorbed by the waste yeast. Winemaking byproducts have shown efficiency in absorbing heavy metals from aqueous solutions. This can be attributed to their concentrations of proteins, carbohydrates, and phenolic compounds, which contain carboxyl, hydroxyl, sulphate, phosphate, and amino groups capable of binding metal ions [102]. Existing research papers generally report low amounts of heavy metals in wine [58,103,104]. According to Orescanin et al. [58], red wine usually contains higher concentrations of metal elements than white wine. Also, there are lower values of heavy metals in wines compared to grapes. Larger amounts of some elements (Fe^2+^, Ni^2+^, Zn^2+^, and Pb^2+^) are associated with contamination during wine storage. The level of some metal ions like Cu^2+^, Fe^2+^, or Mn^2+^ is briefly discussed in several papers. High residual metal content can contribute to the oxidation process, resulting in a loss of aromatic freshness in wines and the precipitation of phenolic compounds in bottled samples. Cabrera-Vique et al. [105] observed a significant increase in chromium content during wine storage. This increase may be attributed to contamination from the stainless steel used for wine storage or the pigmentation of bottles with chromium oxide [106]. The high residual manganese levels favor acetaldehyde formation in wines, which leads to higher polymerization and later precipitation of phenolic compounds. Additionally, when aluminum levels exceed 10 mg/L of Al^2+^ it can cause aluminum cloudiness in wines [107].

Some processes (ion exchange, reverse osmosis, and adsorption) contribute to a significant reduction of metals [2]. Focea et al. [103] reported an increase in heavy metals during the storage of sparkling wines, which was influenced by the evolution of pH, the activation of prosthetic metalloenzymes, and the ionic balance. Andrieș et al. [104] reported a major difference in metal profile when reverse osmosis was applied to obtain low-alcoholic white wines.

According to the presented results, beer and wine do not represent a health risk regarding the content of heavy metals.

**Table 5:** Heavy metals in wine and beer.

Analyzed Product	Country	Identified Heavy Metal	Concentrations	Year	References
Beer	Ethiopia	Cu^2+^	0.0368 mg/L	2022	[2]
		Cd^2+^	0.0014 mg/L		
		Mn^2+^	0.0954 mg/L		
		Pb^2+^	0.006 mg/L		
		Zn^2+^	1.5206 mg/L		
	Italy	Cd^2+^	nd–0.00058 mg/L	2008	[108]
		Pb^2+^	nd–0.0133 mg/L		
	Brazil	Cd^2+^	nd–0.0143 mg/L	2003	[109]
		Pb^2+^	0.013–0.0329 mg/L	2005	[110]
		Zn^2+^	0.0527–0.226 mg/L		
Sparkling wines—Muscat Ottonel	Romania	Mn^2+^	668.84–712.17 µg/L	2017	[103]
		Zn^2+^	216.3–387.4 µg/L		
		Fe^2+^	1214.7–1704.2 µg/L		
		Cu^2+^	111.3–188.1 µg/L		
		Ni^2+^	128.11–169.14 µg/L		
		Pb^2+^	2.46–631.9 µg/L		
White low-alcoholic wine—Muscat Ottonel	Romania	Zn^2+^	2.0–320.1 µg/L	2017	[104]
		Fe^2+^	404.5–1401.5 µg/L		
		Cu^2+^	107.1–726.6 µg/L		
		Mn^2+^	276.2–857.3 µg/L		
		Ni^2+^	58.3–736.2 µg/L		
		Pb^2+^	66.7–574.5 µg/L		
White wine	Croatia	V^2+^	1 µg/L	2008	[58]
		Cr^2+^	3 µg/L		
		Mn^2+^	5 µg/L		
		Fe^2+^	61 µg/L		
		Ni^2+^	6 µg/L		
		Cu^2+^	6 µg/L		
		Zn^2+^	59 µg/L		
		As^3+^	nd		
		Pb^2+^	30 µg/L		

### 4.6. Reduction of Metal Content

Extensive anthropogenic activities, combined with a lack of recycling or proper disposal practices, pose a significant threat to both human life and the environment. Given that most procedures are expensive and logistically challenging, current research efforts are focused on developing cost-effective methods for the removal of contaminants. An overview of the procedures for the reduction of heavy metals in food products is presented below.

Chemical precipitation refers to the administration of some coagulants (lime, alum, and organic polymers) [5]. Pohl [111] highlighted the effectiveness of sulfur-containing precipitation agents in significantly removing several heavy metals (Cd^2+^, Cr^2+^, Co^2+^, Cu^2+^, Hg^2+^, etc.) from water. Chemical reagents like Ca(OH)_2_–calcium hydroxide or NaOH–sodium hydroxide have been used to remove Cu^2+^ ions from aqueous solutions [112,113,114]. This procedure is easy to operate and practicable for most metals, with low costs, but is limited by the high consumption of precipitants and lower removal efficiency, with extra operational costs for sludge disposal [115].

Different electrochemical methods (ion exchange, electrooxidation, electrochemical reduction, and electroflotation) were developed for removing food contaminants [116]. Of these, the ion exchange procedure manifests excellent selectivity for metals but implies high costs with raw materials and a small range of action on metal ions [115].

Electrodialysis is based on the effect of electric potential when a semi-permeable membrane is used to remove metal ions [5]. On this line, Ottosen et al. [117] achieved an 80% reduction of Cu^2+^, Cr^2+^, and As^3+^ from wood wastes after 7 days of treatments with oxalic acid. Also, Bazinet [118] confirmed the efficiency of using electrodialysis with a bipolar membrane in the dairy industry. This method is easy to perform, practicable for most types of metals, and effective for large volumes of samples. The main limitations are represented by the high consumption of electrode materials, secondary pollution, etc. [115].

Coagulation and flocculation processes are effective methods for removing certain metals, and compounds like aluminum, ferrous sulfate, or ferric chloride can be employed for this purpose [5]. The main drawback of this method is its high operational costs. Moreover, large quantities of coagulants and flocculants are necessary to achieve the required level of flocculation [115].

Flotation methods include dissolved air, ions, and precipitation flotation. In the ion flotation process, a surfactant is used to increase the hydrophobicity of the metal ions, which are then removed by air bubbles [5]. The technique is characterized by high efficiency and separation selectivity, but the operation process is complex, with high costs, and the elimination efficiency is reduced [115].

Photocatalytic removal methods refer to the use of light and semiconductors, particularly when applied to wastewater treatment [5]. This technique permits the simultaneous removal of metals, and the resultant by-products present low toxicity. Photocatalytic removal efficiency is limited by the pH range of the medium, involves a long time duration and high energy consumption, and is more difficult to achieve [115].

Adsorption-based processes can be effective in reducing contamination. For this, nanoporous carbon-based adsorbents, chitosan-based adsorbents, or some agricultural wastes can be used (neem leaf, bark, seeds, sugarcane bagasse, rice husk, coconut husk, oil plan shell) [5,119,120,121,122,123,124]. Luchian et al. [123] presented the benefits of Al-MCM-41 mesoporous and mordenite microporous nuclear sieves as sorbents for Mn^2+^, Ni^2+^, and Cu^2+^ in white wines. Adsorption methods are simple, economical, and effective for most metals and involve vast and economic biosorbents. The efficiency of this procedure is dependent on pH (limited pH range), has a long equilibrium time, and is problematic to regenerate [115].

## 5. Conclusions

This review emphasizes existing studies on the content of heavy metals in various food categories, including fruits and vegetables, milk and dairy products, meat and its derivatives, edible oils, and certain alcoholic beverages such as wine and beer. The levels of heavy metals in food are heterogeneous due to the numerous factors that can affect their accumulation. The contamination of food with heavy metals doesn’t pose a risk to human health in most regions. However, there are some values that exceed the limits established by international legislation, especially in countries with large industrial activity or high pollution levels. Still, environmental contamination shows increasing tendencies, and monitoring of heavy metals needs to be continuous to protect human public health. Heavy metal toxicity is directly related to their accumulation in food. High amounts of these elements generate numerous health issues. Therefore, consumers must choose their food sources carefully. Metals like lead, cadmium, and arsenic are the main elements with high potential health risks. According to the analyzed studies, the highest value for cadmium was registered in lettuce cultivated in Romania; excessive amounts of lead were found in apple samples from Ukraine and Kosovo, but also in lettuce or red potatoes from Romania; and arsenic presented alarming values in lettuce from Florida. Reducing their levels and improving food safety require a well-planned approach and the adoption of sustainable agricultural practices, which should be accompanied by efforts to reduce pollution. Given the extensive nature of this subject, more comprehensive studies spanning the entire food chain are imperative.

## Figures and Tables

**Table 1:** Identification of heavy metals in fruits and vegetables.

Food Type	Country	Potential Sources	Identified Heavy Metals	Concentrations	Year	References
Grain, maize	China	Sewage effluent	Cr^2+^	0.08–0.38 mg/kg	2015	[54]
			Pb^2+^	0.02–0.013 mg/kg		
			Cu^2+^	0.16–0.85 mg/kg		
			Zn^2+^	0.16–0.53 mg/kg		
Lettuce	Spain	Air industrialization and vehicles	Ni^2+^	< 0.02 mg/kg	2018	[55]
			Hg^2+^	< 0.008 mg/kg		
			As^3+^	< 0.005 mg/kg		
			Cd^2+^	< 0.005 mg/kg		
	Florida, USA	Different sources	As^3+^	27.3 mg/kg	2017	[56]
	Romania	Intensive agriculture, high pollution	Cd^2+^	0.97–1.52 mg/kg	2021	[62]
			Cu^2+^	8.1–11.8 mg/kg		
			Pb^2+^	0.82–2.22 mg/kg		
			Zn^2+^	59.5–139.5 mg/kg		
Tomato	Romania	Multiple sources	Cu^2+^	32.5–46.0 µg/g	2023	[63]
			Mn^2+^	13.0–13.5 µg/g		
			Fe^2+^	30.5–33.5 µg/g		
			Cd^2+^	nd–0.1 µg/g		
			Pb^2+^	0.65–0.75 µg/g		
			Zn^2+^	4.5–9.5 µg/g		
			Co^2+^	nd–2.2 µg/g		
Radish	India	Multiple sources	Cu^2+^	5.96 mg/kg	2008	[64]
Potato	Egypt	Inadequately treated water	Cu^2+^	0.83 mg/kg	2018	[65]
	China		Cu^2+^	1.03 mg/kg	2009	[66]
			Cr^2+^	0.03 mg/kg		
			Pb^2+^	0.067 mg/kg		
			Cd^2+^	0.015 mg/kg		
	Bangladesh	Multiple sources	As^3+^	0.006 mg/kg	2016	[59]
			Cd^2+^	0.013 mg/kg		
			Pb^2+^	0.007 mg/kg		
			Cr^2+^	0.528 mg/kg		
			Mn^2+^	6.928 mg/kg		
			Ni^2+^	0.643 mg/kg		
			Cu^2+^	4.3 mg/kg		
			Zn^2+^	3.019 mg/kg		
White potato	Romania	Intensive agriculture, high pollution	Cd^2+^	0.13–0.34 mg/kg	2021	[62]
			Cu^2+^	0.1–0.24 mg/kg		
			Pb^2+^	0.3–0.4 mg/kg		
			Zn^2+^	41.0–94.5 mg/kg		
Red potato			Cd^2+^	0.16–0.46 mg/kg		
			Cu^2+^	5.4–6.4 mg/kg		
			Pb^2+^	0.37–1.03 mg/kg		
			Zn^2+^	56.5–64.0 mg/kg		
Grape—white varieties	Croatia	The excessive use of blue vitriol as a fungicide	V^2+^	12–59 ng/g	2008	[58]
			Cr^2+^	18–203 ng/g		
			Mn^2+^	14–180 ng/g		
			Fe^2+^	5–40 ng/g		
			Ni^2+^	1–7 ng/g		
			Cu^2+^	52–533 ng/g		
			Zn^2+^	5–43 ng/g		
			As^3+^	0.3–3.8 ng/g		
			Pb^2+^	1–21 ng/g		
Grape—red varieties	Croatia	The excessive use of blue vitriol as a fungicide	V^2+^	24–63 ng/g		
			Cr^2+^	11–175 ng/g		
			Mn^2+^	82–229 ng/g		
			Fe^2+^	107–204 ng/g		
			Ni^2+^	3–6 ng/g		
			Cu^2+^	55–210 ng/g		
			Zn^2+^	103–207 ng/g		
			As^3+^	0.2–4.9 ng/g		
			Pb^2+^	2–39 ng/g		
Grapes	Egypt	Multiple sources	Cd^2+^	nd	2019	[53]
			Cr^2+^	nd–1.06 mg/kg		
			Cu^2+^	0.72–3.52 mg/kg		
			Pb^2+^	nd		
			Ni^2+^	0.3–1.78 mg/kg		
Banana	Bangladesh	Multiple sources	As^3+^	nd	2016	[59]
			Cd^2+^	nd		
			Pb^2+^	0.003 mg/kg		
			Cr^2+^	0.317 mg/kg		
			Mn^2+^	10.74 mg/kg		
			Ni^2+^	0.037 mg/kg		
			Cu^2+^	0.946 mg/kg		
			Zn^2+^	0.235 mg/kg		
Mango	Bangladesh	Multiple sources	As^3+^	0.013 mg/kg	2016	[59]
			Cd^2+^	0.005 mg/kg		
			Pb^2+^	0.642 mg/kg		
			Cr^2+^	0.893 mg/kg		
			Mn^2+^	6.06 mg/kg		
			Ni^2+^	0.293 mg/kg		
			Cu^2+^	7.891 mg/kg		
			Zn^2+^	0.604 mg/kg		
Onion	Romania	Intensive agriculture, high pollution	Cd^2+^	0.17–0.34 mg/kg	2021	[62]
			Cu^2+^	3.6–12.3 mg/kg		
			Pb^2+^	0.16–0.18 mg/kg		
			Zn^2+^	28.0–47.0 mg/kg		
Carrot	Romania	Intensive agriculture, high pollution	Cd^2+^	0.27–0.69 mg/kg	2021	[62]
			Cu^2+^	6.0–9.4 mg/kg		
			Pb^2+^	0.54–0.94 mg/kg		
			Zn^2+^	51.0–75.5 mg/kg		
Beans	Romania	Intensive agriculture, high pollution	Cd^2+^	0.04–0.05 mg/kg	2021	[62]
			Cu^2+^	7.0–11.1 mg/kg		
			Pb^2+^	0.08–0.52 mg/kg		
			Zn^2+^	58.5–91.0 mg/kg		
Apples	Kosovo	Intensive mining and smelting activities	Pb^2+^	1.49–2.17 mg/kg	2019	[67]
			Cd^2+^	0.17–0.21 mg/kg		
			Cr^2+^	8.22–11.6 mg/kg		
			Ni^2+^	15.33–17.2 mg/kg		
			As^3+^	nd–0.009 mg/kg		
			Zn^2+^	1.51–2.03 mg/kg		
			Cu^2+^	1.05–2.87 mg/kg		
			Fe^2+^	2.27–3.12 mg/kg		
	Egypt	Multiple sources	Cd^2+^	nd	2019	[53]
			Cr^2+^	nd–1.06 mg/kg		
			Cu^2+^	0.72–3.52 mg/kg		
			Pb^2+^	nd		
			Ni^2+^	0.3–1.78 mg/kg		
	Ukraine	Nature management conflicts; intensive heavy industry	Cr^2+^	0.0225–0.246 mg/kg	2021	[59]
			Zn^2+^	0.964–4.192 mg/kg		
			Cu^2+^	0.766–1.264 mg/kg		
			Cd^2+^	0.088–0.284 mg/kg		
			Pb^2+^	1.347–3.886 mg/kg		
Oranges	Egypt	Multiple sources	Cd^2+^	nd	2019	[53]
			Cr^2+^	nd		
			Cu^2+^	0.36–2.2 mg/kg		
			Pb^2+^	nd		
			Ni^2+^	0.06–0.38 mg/kg		

**Table 2:** Evaluation of heavy metals in milk and dairy products.

Analyzed Product	Country	Identified Heavy Metal	Concentrations	Year	References
Raw cow milk	Egypt	Hg^2+^	0.0014 mg/kg	2023	[68]
		As^3+^	0.012 mg/kg		
		Pb^2+^	0.1016 mg/kg		
		Cd^2+^	0.07 mg/kg		
		Cr^2+^	0.1044 mg/kg		
		Cu^2+^	0.0656 mg/kg		
Milk and dairy products	Egypt	Pb^2+^	0.044–0.751 ppm	2014	[69]
		Cd^2+^	0.008–0.179 mg/kg		
		Zn^2+^	0.888–18.316 mg/kg		
		Cu^2+^	0.002–1.692 mg/kg		
Milk	Monte Carlo	Pb^2+^	0.55 mg/kg	2023	[73]
		Cd^2+^	0.003 mg/kg		
Raw cow milk	Turkey	Pb^2+^	16.7 μg/kg	2012	[70]
		Cd^2+^	0.53 μg/kg		
		Hg^2+^	0.18 μg/kg		
		As^3+^	4.02 μg/kg		
Milk	Turkey	As^3+^	1.01 μg/kg	2023	[61]
		Al^2+^	71.89 μg/kg		
		Ni^2+^	98.53 μg/kg		
		Cd^2+^	nd		
		Pb^2+^	0.85 μg/kg		
Full fat UHT milk	Cyprus	As^3+^	2.33 μg/kg	2021	[62]
		Cd^2+^	5.00 μg/kg		
		Pb^2+^	2.66 μg/kg		
		Hg^2+^	3.66 μg/kg		
Halloumi cheese		As^3+^	12.33 μg/kg		
		Cd^2+^	44.33 μg/kg		
		Pb^2+^	35.33 μg/kg		
		Cu^2+^	591.33 μg/kg		
		Hg^2+^	15.00 μg/kg		
Full-fat yogurt		As^3+^	6.00 μg/kg		
		Cd^2+^	36.33 μg/kg		
		Pb^2+^	3.00 μg/kg		
		Cu^2+^	15.66 μg/kg		
		Hg^2+^	6.00 μg/kg		
Cream with a minimum of 10% milk fat	Republic of Srpska and Bosnia and Herzegovina	As^3+^	0.019 mg/kg	2023	[52]
Cream with over 70% milk fat		As^3+^	0.029 mg/kg		
Cheese		As^3+^	0.012–0.015 mg/kg		
Milk	Tanzania	Pb^2+^	0.263 mg/kg	2023	[74]
		Cd^2+^	nd		
		Co^2+^	0.020 mg/kg		
Sheep and goart milk	Italy	As^3+^	nd	2020	[75]
		Cd^2+^	nd		
		Hg^2+^	nd		
		Ni^2+^	nd		
		Pb^2+^	0.002 μg/g		
		Zn^2+^	2.77 μg/g		

**Table 3:** Heavy metals levels in meat and meat derivatives.

Analysed Product	Country or Regions	Identified Heavy Metal	Concentrations	Year	References
Pork meat products	Italy	Cr^2+^	0.15–0.23 mg/kg	2020	[76]
		Cd^2+^	0.01–0.03 mg/kg		
		Hg^2+^	0.01–0.02 mg/kg		
		Cu^2+^	1.08–1.21 mg/kg		
		Pb^2+^	0.22–0.38 mg/kg		
Beef meat	Iran	Cd^2+^	0.028 mg/kg		
		Hg^2+^	0.003 mg/kg		
Bacon	Romania	Pb^2+^	0.58 mg/kg	2014	[77]
		Cd^2+^	0.11 mg/kg		
		Cu^2+^	1.02 mg/kg		
		Zn^2+^	42.1 mg/kg		
Mutton meat	China (Beijing)	Cr^2+^	0.654 mg/kg	2019	[79]
		Cd^2+^	0.031 mg/kg		
		Pb^2+^	0.128 mg/kg		
		As^3+^	0.008 mg/kg		
		Hg^2+^	0.005 mg/kg		
Ham	Romania	Pb^2+^	0.65 mg/kg	2014	[77]
		Cd^2+^	0.13 mg/kg		
		Cu^2+^	0.73 mg/kg		
		Zn^2+^	33.5 mg/kg		
Salami	Romania	Pb^2+^	0.96 mg/kg	2014	[77]
		Cd^2+^	0.21 mg/kg		
		Cu^2+^	1.32 mg/kg		
		Zn^2+^	32.19 mg/kg		
Sausage	Romania	Pb^2+^	0.82 mg/kg	2014	[77]
Beef meat	Republic of Srpska and Bosnia and Herzegovina	As^3+^	0.005–0.030 mg/kg	2023	[52]
		Cd^2+^	0.001–0.012 mg/kg		
Pork meat		As^3+^	0.005–0.022 mg/kg		
		Cd^2+^	0.001–0.007 mg/kg		
Poultry meat		As^3+^	0.006–0.015 mg/kg		
		Cd^2+^	0.001 mg/kg		
Mechanically debonet meat		As^3+^	0.05–0.016 mg/kg		
		Cd^2+^	0.001–0.002 mg/kg		
Red meat	Asia	Pb^2+^	605.3–1435.18 μg/kg	2023	[80]
		Cd^2+^	206.45–257.79 μg/kg		
	Africa	Pb^2+^	840.64–1094.42 μg/kg		
		Cd^2+^	74.69–94.66 μg/kg		
Beef	Italy	As^3+^	0.012 μg/g	2020	[75]
		Cd^2+^	nd		
		Hg^2+^	nd		
		Ni^2+^	nd		
		Pb^2+^	0.019 μg/g		
		Zn^2+^	48.94 μg/g		
Pork		As^3+^	0.015 μg/g		
		Cd^2+^	nd		
		Hg^2+^	nd		
		Ni^2+^	nd		
		Pb^2+^	0.024 μg/g		
		Zn^2+^	44.91 μg/g		

**Table 4:** The presence of heavy metals in edible oils.

Analyzed Product	Country	Identified Heavy Metal	Concentrations	Year	References
Corn oil	Turkey	Cd^2+^	0.0012 mg/kg	2008	[84]
		Ni^2+^	0.0015 mg/kg		
		Zn^2+^	0.0330 mg/kg		
	Iran	Pb^2+^	0.099 mg/kg	2020	[89]
Soybean oil	Turkey	Cd^2+^	0.0013 mg/kg	2008	[84]
		Ni^2+^	0.0027 mg/kg		
		Zn^2+^	0.0348 mg/kg		
	Salvador, Brazil	Cu^2+^	0.83 mg/kg	2015	[85]
Rice oil	Illnios, USA	Cd^2+^	<0.891 mg/kg	2015	[86]
Olive oil	Cyprus	Cd^2+^	0.05 mg/kg	2019	[87]
	Turkey	Cd^2+^	0.9922 mg/kg	2021	[88]
	Iran	Cd^2+^	0.0955 mg/kg	2020	[89]
	London	Pb^2+^	0.143 mg/kg	2022	[94]
	Pakistan	Pb^2+^	4.285 mg/kg	2022	[95]
	Ukraine	Cu^2+^	0.355 mg/kg	2015	[96]
	Greece	Cu^2+^	0.086 mg/kg	2022	[97]
Rapeseed oil	Iran	Cd^2+^	0.098 mg/kg	2020	[89]
	China	Pb^2+^	1.96 mg/kg	2016	[90]
	Poland	Pb^2+^	0.056 mg/kg	2017	[91]
	Salvador, Brazil	Cu^2+^	0.81 mg/kg	2015	[85]
Sesame oil	Iran	Cd^2+^	0.0935 mg/kg	2020	[89]
Coconut oil	London	Pb^2+^	0.158 mg/kg	2022	[94]
Sesame oil	Pakistan	Pb^2+^	4.005 mg/kg	2022	[95]
Sunflower oil	London	Pb^2+^	0.274 mg/kg	2022	[94]
	Iran	Pb^2+^	0.099 mg/kg	2020	[89]
	Salvador, Brazil	Cu^2+^	0.81 mg/kg	2015	[85]
Linseed oil	Poland	Cu^2+^	0.10 mg/kg	2016	[98]
Flaxseed oil	Korea	Pb^2+^	25.65 µg/kg	2019	[41]
		Cd^2+^	70.03 µg/kg		
		As^3+^	3.1 µg/kg		
		Al^2+^	29.81 mg/kg		
Sesame oil		Pb^2+^	36.01 µg/kg		
		As^3+^	15.18 µg/kg		
		Al^2+^	15.97 mg/kg		
Canola	Monte Carlo	As^3+^	0.062–0.118 mg/kg	2022	[92]
		Cd^2+^	0.097–0.123 mg/kg		
		Cu^2+^	0.027–0.041 mg/kg		
Corn		As^3+^	0.095–0.106 mg/kg		
		Cd^2+^	0.018–0.045 mg/kg		
		Cu^2+^	0.027–0.041 mg/kg		
		Ni^2+^	0.024–0.076 mg/kg		
		Zn^2+^	0.613–1.090 mg/kg		
Cottonseed		As^3+^	0.104–0.116 mg/kg		
		Cd^2+^	0.001–0.008 mg/kg		
		Cu^2+^	0.010–0.062 mg/kg		
		Ni^2+^	0.016–0.078 mg/kg		
		Pb^2+^	−0.004–0.016 mg/kg		
		Zn^2+^	−0.060–2.148 mg/kg		
Soybean		As^3+^	−0.037–0.125 mg/kg		
		Cd^2+^	0.058–0.129 mg/kg		
		Cu^2+^	0.247–0.283 mg/kg		
		Ni^2+^	0.579–0.677 mg/kg		
		Pb^2+^	0.033–0.070 mg/kg		
		Zn^2+^	0.357–0.588 mg/kg		
Sunflower		As^3+^	−0.019–0.147 mg/kg		
		Cd^2+^	0.024–0.038 mg/kg		
		Cu^2+^	−0.026–0.233 mg/kg		
		Ni^2+^	0.153–0.221 mg/kg		
		Pb^2+^	0.022–0.025 mg/kg		
		Zn^2+^	0.411–0.456 mg/kg		
